# One for the road! A study to assess the efficacy of single low-dose regimen of rasburicase in controlling hyperuricaemia in patients with tumour lysis syndrome due to haematological malignancies

**DOI:** 10.3332/ecancer.2013.378

**Published:** 2013-12-10

**Authors:** Hamdy A Azim, Sherif Ahmed Bahr, Nermine Shawky Kamal, Mohamed Adel Koura, Rehab Tolba, Heba Abdelmoneem Gad, Ahmad Morsy, Hossameldin Mohsen Attia, Ibraheem Iskander, Ahmed Hammad, Mohammed Farouk Hemed, Mohammed Fathy Abdallah, Kareem Ahmed Sadek, Alaa Hamdi Taha

**Affiliations:** 1 Department of Clinical Oncology, Faculty of Medicine, Cairo University, Cairo, Egypt; 2 Clinical Oncology and Bone Marrow Transplantation Unit, Manial Specialized University Hospital, Cairo University, Cairo, Egypt

**Keywords:** tumour lysis syndrome, hyperuricaemia, single dose of rasburicase, haemological malignancies

## Abstract

We conducted a retrospective audit of six patients with various haematological malignancies (two acute lymphoblastic leukaemia, one acute myeloid leukaemia, and three non-Hodgkin lymphoma); these patients were eligible to receive rasburicase, being at high risk of development of tumour lysis syndrome (TLS). They received a fixed single low-dose regimen of rasburicase (7.5 mg) mainly due to financial restriction, as patients were not supported by the National Health Service and did not have health insurance. We compared uric acid, creatinine levels, and electrolytes (i.e. phosphate, potassium, and calcium) before and after rasburicase administration and also assessed the need for renal replacement therapy after treatment.

All six patients had a significant reduction in uric acid levels on the first day, achieving a response rate of 100% (*p *= 0.008994); creatinine, phosphate, and potassium were reduced significantly as well, with the *p *values of 0.0439, 0.014326, and 0.002008, respectively; only one patient needed renal replacement therapy in the form of haemodialysis, due to concerns about hyperphosphataemia.

Financial difficulties faced either because patients lacked insurance or because of the restricted National Health Service budget in Egypt have resulted in the unavailability of certain modalities of treatment in cancer care and the need to consider more economic yet efficient approaches. Our experience suggests that a single low-dose rasburicase injection (7.5 mg) is an efficient and cost-effective method to control hyperuricaemia in patients with a high risk of developing TLS when compared with the more expensive and extended standard regimen and doses recommended.

## Introduction

Tumour lysis syndrome (TLS) is an overwhelming oncologic emergency that can result in serious consequences and requires prompt intervention. A great advance in the management of the condition has been achieved by adding rasburicase to the existing arsenal used to prevent and treat this condition, but the recommended regimen and doses can pose a financial challenge for patients with no insurance as well as being affected by the restricted budgets available for National Egyptian Health Services. With data and studies suggesting similar efficacy for abbreviated courses and doses of rasburicase in the management of TLS, there was a need to assess that strategy in a cohort of Egyptian patients to achieve a more economic approach while achieving reasonably comparable standards of efficiency, efficacy, and safety.

We conducted a retrospective case series reporting on six patients who received a fixed single low-dose regimen of rasburicase (7.5 mg) at high risk of developing TLS due to various haemotological malignancies. Uric acid and creatinine levels as well as the need for haemodialysis after rasburicase administration were analysed.

Our experience suggests that a single low-dose rasburicase injection (7.5 mg) seems to be an efficient and cost-effective method to control hyperuricaemia in patients at high risk of developing TLS.

## Methods

### Study objective

To evaluate single fixed dosing of 7.5 mg rasburicase in terms of controlling hyperuricaemia and subsequently serum creatinine in the treatment or prevention of TLS in patients with haematological malignancies, the primary end point is control of serum uric acid levels on days 1, 2, and 3 as well as on day 7 of follow-up to emphasise the maintenance of that control and the secondary end point is control of serum creatinine, serum phosphate, and serum potassium levels on days 1, 2, and 3 as well as on day 7 of follow-up.

## Patients

During the period between May 2011 and July 2012, six patients within an age range of 18–50 years (mean = 35.5 years, SD = 10.49) at high risk of developing TLS due to various haematological malignancies, that is, non-Hodgkin lymphoma (NHL – 3), acute lymphoblastic leukaemia (ALL – 2), and acute myeloid leukaemia (AML – 1), were treated at the Clinical Oncology departments at Cairo University Hospitals by single low-dose rasburicase on seven occasions (one patient experienced high risk of TLS upon his initial presentation and upon disease relapse). Most patients bore a high tumour burden that was assumed by high total leukocytic counts (TLCs) that contributed to a high risk of TLS; two of seven patients had notable lymphadenopathy, but none had a bulky tumour mass (i.e., all masses noted were less than 10 cm in maximum diameter); none of the patients had a history or any signs suggestive of end-stage renal disease or chronic kidney disease. Upon presentation, all patients were hyperuricaemic, and five of the seven patients had creatinine levels > 1.5 × ULN with high risk of acute renal injury. The patients’ characteristics are shown in Table 1, and baseline laboratory findings in each patient are shown in Tables 2–6.

## Patient evaluation

According to the current guidelines, patients with haematological malignancies at risk of development of TLS are evaluated at baseline for TLC, creatinine, and uric acid levels together with electrolytes, that is, potassium, calcium, and phosphorus. Follow-up lab work was done at least daily for the same parameters.

## Statistical analysis

All patients were included in the efficacy and safety analyses; response was defined as decline of serum uric acid levels below the threshold for hyperuricaemia and maintenance of this decline through days 1–7 of therapy. The improvement of renal functions was assessed by following the decline of serum creatinine levels and maintenance of this decline throughout days 1–7 of therapy; the decline in serum phosphate and serum potassium was also assessed throughout the 7 days of follow-up. Pre-treatment and post-treatment serum uric acid, serum creatinine, serum phosphate, and serum potassium levels were compared using a paired *t* test using Microsoft Excel software.

Adverse events were graded according to the World Health Organization toxicity criteria.

## Treatment modalities

A total of six patients received rasburicase as a 30-minute intravenous infusion, at a fixed dose of 7.5 mg, in 50 mL preservative-free sodium chloride (range 0.083–0.11 mg/kg, mean 0.095 mg/kg, SD 0.01) on seven occasions (one patient experienced high risk of TLS upon his initial presentation and upon disease relapse). Patients only received treatment with rasburicase once with no subsequent injections planned unless clinically indicated, and none more were needed to any of the patients; all patients received intravenous hydration at 3–4 L/day monitored by their jugular venous pressure, and five of seven patients were treated with concomitant allopurinol at a dose of 300 mg orally twice daily, as shown in Table 7.

This study has been conducted in line with local regulations at the University of Cairo Hospital’s oncology department and after being approved by the institutional review board.

## Results

### Efficacy

The primary end point of a significant decrease in plasma uric acid levels on the first day following treatment with fixed single low dose of rasburicase was met with a *p *value of 0.008994 with a sustained response maintained through days 2 and 3 of follow-up. The response rate reached 100% as all patients’ uric acid levels normalised at day 4 of therapy. The significant reduction of uric acid levels at day 7 of therapy with no need for further treatment suggests the efficacy of the single-low dose approach.

Only one patient needed to undergo haemodialysis due to high phosphate levels rather than uric acid that were reduced, and there was no need to postpone chemotherapy or reduce chemotherapy doses in this patient. A significant reduction in plasma creatinine levels on the first day after treatment with a *p *value of 0.0439, which showed further decline in all patients on days 2 (*p *= 0.01299781) and 3 (*p *= 0.007507) of treatment up to day 7 (*p *= 0.018375), reflects a decreased incidence of acute renal failure requiring haemodialysis for management, thus meeting this secondary end point.

Secondary end points of significant reduction in serum phosphate and serum potassium levels on day 1 of follow-up post-treatment have also been met with *p *values of 0.014326 and 0.002008, respectively, a response that was also maintained throughout days 2–7 of follow-up.

### Safety

All patients received treatment with good tolerability, and no grade 3 or 4 adverse events were recorded that were attributable to rasburicase administration. No cases of allergic reactions or haemolytic anaemia were recorded.

This study was conducted in line with local regulations at the University of Cairo Hospital’s oncology department and after being approved by the institutional review board.

## Discussion

TLS is an oncologic emergency that can occur due to any type of neoplasm; it is caused by a massive lysis of tumour cells disturbing renal function, electrolytes, and haemodynamics and requiring immediate intervention.

Uric acid nephropathy due to mechanical obstruction by uric acid crystals in the renal tubules is the major cause of acute renal failure in the setting of TLS enhanced by high acidity and high concentration in the renal tubular fluid. Renal medullary haemoconcentration and decreased tubular flow rate also contribute to crystallisation [1].

Acute nephrocalcinosis due to calcium phosphate crystal precipitation, which may occur in other tissues, develops in the setting of hyperphosphataemia and is exacerbated by overzealous iatrogenic alkalinisation, because calcium phosphate, unlike uric acid, becomes less soluble at an alkaline pH.

TLS is highly associated with rapidly proliferating and bulky tumours compared with tumours of small volume or small tumour burden; Hande and Garrow showed in 1993 an incidence of TLS of 42% in a cohort of 102 patients with high-grade NHL most commonly in ALL and high-grade NHL, in particular Burkitt’s lymphoma. Other haematological malignancies that have a lower incidence of TLS include chronic lymphocytic leukaemia, AML, and plasma cell disorders including multiple myeloma and isolated plasmacytomas. In addition, TLS has been reported with other haematological malignancies including low-grade and intermediate-grade NHL, Hodgkin’s disease, chronic myeloid leukaemia (CML) in blast crisis, and myeloproliferative disorders [2].

In the context of treating patients presenting with high-risk features of developing TLS including hyperuricaemia due to highly proliferative neoplasms, especially haematological malignancies; an alternative to inhibiting uric acid formation by inhibiting xanthine oxidase with allopurinol is to promote the catabolism of uric acid to allantoin by urate oxidase. In comparison with uric acid, allantoin is five to ten times more soluble in urine [3], also avoiding accumulation of xanthine and hypoxanthine, which demonstrate poor water solubility and can worsen renal function [4]. Urate oxidase is an endogenous enzyme commonly found in many mammalian species but not in humans, secondary to a nonsense mutation in the coding region during hominoid evolution [5]. A non-recombinant urate oxidase, extracted from *Aspergillus flavus*, has been demonstrated to reduce uric acid levels in patients at risk for TLS and has been available in France since 1975 and in Italy since 1984 [6–8]. Recently, the gene coding for the urate oxidase was isolated as a cDNA clone from the *A. flavus *and expressed in the yeast *Saccharomyces cerevisiae *to yield large quantities of the pure recombinant form of urate oxidase (rasburicase) [9]. Pui *et al *initially reported the prophylactic use of rasburicase (15–20 mg/kg i.v. q.i.d. for 5–7 d) in 66 children with haematological malignancies at risk for TLS who presented with normouricaemic levels (uric acid <476 Lmol/L) [10]. Pui *et al *demonstrated a significant reduction of the median uric acid level of 256–30 Lmol/L (*p* < 0001) within 4 hours [10].

The phase III study results published by Cortes *et al *demonstrated efficacy and safety of rasburicase alone or followed by allopurinol compared with allopurinol alone in controlling plasma uric acid in adults at risk for TLS [11]; the results of the GRAAL1 (Grouped’Etude des Lymphomes de l’Adulte Trial on Rasburicase Activity in Adult Lymphoma) study have reached similar results before while assessing patients with aggressive NHL during induction treatment [12].

However, optimal dosage and timing of rasburicase should be discussed. Although the European Medicines Agency and the US Food and Drug Administration recommend a dosing range of 0.15 to 0.2 mg/kg/d for 5 days for rasburicase, several studies have demonstrated that a shorter treatment period or a lower dosage might have similar efficacy and might be cost saving [13–25]; one is a report published by Ho *et al *who demonstrated the efficacy of abbreviated doses of rasburicase in patients with high risk of developing TLS [23].

Another report by McBride *et al *concluded that the efficacy of all single fixed doses and weight-based dosing strategies evaluated in the study (3, 6, and 7.5 mg) appear to be comparable in normalising plasma uric acid levels within 24 hours of rasburicase administration, and although the use of a 3-mg rasburicase dose may be the most cost-effective treatment strategy in managing hyperuricaemia secondary to TLS, the 6-mg dose resulted in lower sustained uric acid levels after rasburicase administration [24].

Rasburicase is contraindicated in pregnancy and in patients with glucose-6-phosphate dehydrogenase (G6PD) deficiency.

## Conclusion

In this case series, we report on the efficacious use of single low-dose rasburicase injection in the control of serum uric acid levels in six patients with haematological malignancies at high risk of developing TLS all through seven days of follow-up post-treatment, significant reduction of uric acid levels (*p *= 0.008994, *p *= 0.003976, *p *= 0.00166, and *p *= 0.003399) at days 1, 2, 3, and 7 of therapy, respectively, with a response rate reaching 100% in all patients.

In addition, other biologic parameters involved in TLS were also controlled, demonstrating once again that the dramatic reduction of uric acid levels is the most important parameter for the prevention of TLS.

Renal function was almost preserved with significant reduction in serum creatinine levels (*p *= 0.043906828, *p *= 0.01299781, *p *= 0.007507, and *p *= 0.018375) on days 1, 2, 3, and 7, respectively, only one patient requiring haemodialysis for acute renal failure due to high levels of serum phosphate rather than high serum uric acid levels, which is of paramount importance in the management of these patients and for the prevention of many complications of chemotherapy. Rasburicase was excellently tolerated as well.

There has been significant reduction of serum phosphate (*p *= 0.014326, *p *= 0.010934, *p *= 0.008864, and *p *= 0.010825) for days 1, 2, 3, and 7 respectively, and serum potassium levels (*p *= 0.002008, *p *= 0.006224, *p *= 0.0069915, and *p *= 0.0044933) for days 1, 2, 3, and 7, respectively, as well.

From an economic point of view, the promising single low-dose approach of rasburicase administration achieved satisfactory results with a great reduction of cost per patient (approximately 3000 LE for the single low-dose approach versus 6000–42000 LE for the standard approved regimen), which can result in better cost effectiveness achieved by physicians caring for such patients with no compromise of clinical efficacy.

The small number of our sample of patients is regarded as a limitation to our study and shows the need for a study on a larger scale to emphasise our results.

## Conflicts of Interest

The authors declare that there are no conflicts of interest.

## Figures and Tables

**Table 1.** Patients’ characteristics.

Number	6
Age (in years)	
Range	18–50
Mean	35.57
Median	37
SD	10.49
Male\female	4\2
Diagnosis	
ALL	2
AML	1
NHL	3
TLC at presentation	
Range (×10^3^/μL)	2.5–200
Mean (×10^3^/μL)	54.93
Median (×10^3^/μL)	25
SD (10^3^/μL)	71
Uric acid at presentation (mg/dL)	
Range (mg/dL)	10.2–61
Mean (mg/dL)	28.6
Median (mg/dL)	18.5
SD (mg/dL)	21.43

SD: standard deviation, ALL: acute lymphoblastic leukaemia, AML: acute myeloid leukaemia, NHL: non-Hodgkin lymphoma, TLC: total leukocytic count.

**Table 2.** Baseline laboratory findings before rasburicase administration.

Patient	Creatinine	Uric acid	PO_4_	K	Ca	TLC
1	6.3	28	14	5.5	7.9	13
2	2.5	15	8.5	5.3	8	10
3	2	20	6.3	5.6	8.8	22
4	5.6	11.5	18	5.6	8	8
4	5.1	28	6	5.5	8.6	185
5	10.5	49	16	6.8	5.2	1.8
6	4	10.2	6.4	3.9	10.8	34

PO_4_: phosphate, K: potassium, Ca: calcium, TLC: total leukocytic count.

**Table 3.** Laboratory findings on day 1 follow-up.

Patient	Day 1 follow-up					
	Creatinine	Uric acid	PO_4_	K	Ca	TLC
1	5.9	24	9	5.2	8	12.5
2	2.7	9	6.7	5	8.3	9.6
3	1.9	9	5.5	5.3	9	18
4	3	9.5	9.9	4.8	7.8	2.2
4	4.2	2.5	4.5	5	9	74
5	10	25	13	5.7	6.5	1.2
6	3.4	1.8	5.9	3.5	10.5	26

PO_4_: phosphate, K: potassium, Ca: calcium, TLC: total leukocytic count.

**Table 4.** Laboratory findings on day 2 follow-up.

Day 2 follow-up						
Patient	Creatinine	Uric acid	PO_4_	K	Ca	TLC
1	5.2	18	6.5	5	8.5	12
2	2	4	5.2	4.5	8.9	7
3	1.9	6	5.3	4.9	9.3	14
4	1.7	9	6	4.9	8	1.4
4	2.9	1.4	4.5	4.7	9	48
5	9.5	19	9	5.5	7.9	2
6	2.9	1.4	5	4.2	9.8	9

PO_4_: phosphate, K: potassium, Ca: calcium, TLC: total leukocytic count.

**Table 5.** Laboratory findings on day 3 follow-up.

Day 3 follow-up						
Patient	Creatinine	Uric acid	PO_4_	K	Ca	TLC
1	5.2	9.5	5.5	4.5	8.6	11
2	1.9	0	5	4.6	9	7.3
3	1.5	2	5	4.5	9	12
4	1.4	6	5.3	4.5	9.6	1.9
4	3	0.9	4.5	4.5	9.3	24
5	7.2	13	6.3	5.2	8.5	3.2
6	3	1	4.2	2.9	10	3.7

PO_4_: phosphate, K: potassium, Ca: calcium, TLC: total leukocytic count.

**Table 6.** Laboratory findings on day 7 follow-up.

Patient	Day 7 follow-up					
	Creatinine	Uric acid	PO_4_	K	Ca	TLC
1	2.9	1.5	4.6	4.7	9.5	7
2	0.8	2	4.5	4.7	9.4	7.8
3	1.2	3	4.8	5	9.5	6.8
4	0.9	2	4.8	4.7	9.2	2.2
4	3.5	2	5	4.9	9.2	2.8
5	1.4	6.1	3.6	3.6	6	2.5
6	1.2	5.9	4.5	4.5	9	0.4

PO_4_: phosphate, K: potassium, Ca: calcium, TLC: total leukocytic count.

**Table 7.** Treatment modalities.

Dose of rasburicase (mg)	7.5
Range (mg/kg)	0.083–0.11
Mean (mg/kg)	0.095
Median (mg/kg)	0.095
SD (mg/kg)	0.01
Allopurinol administered	5 of 7

SD: standard deviation.
